# Progress, Challenges, and Opportunities of Geriatric Medicine in China

**DOI:** 10.14336/AD.2022.0128

**Published:** 2022-10-01

**Authors:** Zeyu Zhao, Xunyao Hou, Shangbin Li, Wenwen Xu, Qingqing Yin

**Affiliations:** ^1^Department of Neurology, The First Affiliated Hospital of Shandong First Medical University & Shandong Provincial Qianfoshan Hospital, Jinan, Shandong, China.; ^2^Department of Geriatric Neurology, Shandong Provincial Hospital Affiliated to Shandong First Medical University, Jinan, Shandong, China.; ^3^Department of Geriatrics, Shandong Provincial Hospital Affiliated to Shandong First Medical University, Jinan, Shandong, China.; ^4^Department of Ophthalmology,Shandong Provincial Hospital Affiliated to Shandong First Medical University, Jinan, Shandong, China

**To the editor,**

Geriatric diseases are among the leading causes of death and major health concerns in modern China. By the end of 2020, there were 255 million people aged 60 years or older in China, accounting for 18.7% of the total population. Among them, 176 million are aged 65 years or older, accounting for 11.97% of the total population. More than 180 million elderly people suffer from chronic diseases, and 75% suffer from one or more chronic diseases [1]. Approximately 40 million elderly people are disabled or partially disabled. Geriatric chronic diseases such as cardiovascular and cerebrovascular diseases and malignant tumors have become the main causes of death, accounting for 87% of all deaths in China (www.gov.cn/guoqing/2021-07/22/content_5626526.htm). Therefore, China has become the country with the largest aged population in the world as well as one of the countries with the fastest aging rate. It is facing severe challenges in physical health care, disease prevention, treatment housing environments, economic support for patients, and social services.

Geriatric medicine is a new comprehensive medical specialty that studies the mechanism and principle of human aging, as well as the occurrence, development, and prevention of senile diseases, and the sociology and other problems related to the physical and mental health of the elderly. Research on aging in China has mainly focused on the epidemiology of common chronic diseases and geriatric syndromes, including physical and cognitive frailty, sarcopenia, multimorbidity, disability and dementia [2,3]. The beginning of modern geriatrics in China is similar to that in the world. In the mid-1950s, the Chinese government proposed revitalizing geriatric medicine and using modern scientific methods to study gerontology. The Ministry of Health established a special committee on geriatrics in 1980 and the Geriatrics Branch of the Chinese Medical Association was established in 1981, the Chinese Geriatrics Journal was published in 1982, and the National Geriatrics Leading Group was established in 1995 [4]. To date, the National Natural Science Fund, National Key Research and Development Program, and National “9^th^ Five-year Plan” to “14^th^ Five-year Plan” attack topics have all included geriatric items. In addition, China established the National Clinical Center for Geriatric Diseases and a standardized training system for geriatric specialists in 2018. In 2019-2021, many provinces established Provincial Clinical Centers for Geriatric Diseases. In 2018, only 25% of general hospitals above primary level in Shandong province had a geriatric medical department (www.shandong.gov.cn/art/2020/2/26/art_100058_8865394.html). More recently, geriatric medical departments have been strengthened and expanded. Under the elderly health promotion agenda of the Healthy China 2030 policy, this proportion will reach 65% by 2022 and 90% by 2030, which will greatly promote the development of geriatrics in China [1].

At present, the vast majority of geriatric medicine in China’s medical institutions originates from the health care department for retired government officials. There is still a considerable divide between geriatrics in China and advanced countries in the world. In long-term chronic care management, levels of awareness, treatment, and control for common diseases such as hypertension, hyperlipidemia, diabetes, cardiovascular and cerebro-vascular disease among the elderly are very low. This is because the number of medical institutions for all the elderly is seriously insufficient, and the coverage and diagnosis capacity of community hospitals and clinics are inadequate, especially those in rural settings. Another notable problem is a weakness in the long-term care structure, especially the shortage of nursing homes and medical personnel specializing in geriatrics. Additionally, geriatric medicine as an independent discipline is not reflected in national residency program training established in 2014 and the current medical school curriculum. Most geriatric doctors in general hospitals come from internal medicine or cardiovascular specialties, so the prevailing medical practice is single-disease-based, while little thought is given to multimorbidity, and comprehensive geriatric assessment is not routinely conducted [5]. Finally, the divide between regions at different developmental levels and between cities and rural areas still exists in China. A few major and central cities have built large numbers of nursing homes through various models including public and private. In contrast, elderly individuals with long-term care needs in poor regions and the countryside largely depend on family care. Ultimately, the government may have to invest far more financial resources to close the gap and improve overall health care access and quality for the elderly and other population groups [4].

The severe challenge of aging in China has attracted great attention from society as a whole, and governments at all levels have significantly strengthened their support and provided unprecedented opportunities for the development of geriatrics. According to the strategic health policy of Healthy China 2030, older people should be provided with health care and geriatric services that include hospitalization during treatment periods, nursing during rehabilitation, daily care during the stable periods, and hospice care [6]. For geriatric medicine departments, comprehensive evaluation will not only help medical personnel understand and grasp the patients’ physical and mental state early on and formulate reasonable intervention measures, but also help medical services and social security departments of different elderly individuals make accurate medical services and support, with minimal interventions to help the elderly to maintain their ability to live independently or assist in life. Because of economic needs and the size of an aging population, home-based and community-supported aged care systems are the way forward. For elderly patients with multiple health problems, continuity of care services is essential, and most services should be provided in the community. The lack of nursing facilities is likely to persist for years, but many community hospitals and clinics are currently underutilized and can potentially divert excess capacity to services for the elderly. Designated major hospitals can form partnerships with community service providers for knowledge transfer and staff training to complement this approach [4]. For high-risk and ill elderly patients, especially for those with end-stage diseases such as late cancer of the elderly, the principle of reducing medical intervention to “maintain life, the life process of death as a normal, neither accelerating or delaying death” and to provide patients with dignity, be prepared, comfortable and quiet, has had increasing approval [7]. People’s understanding of the quality of death can be improved by means of conference education and collective discussion.

China has realized the health and social challenges of population aging and has given high priority to overcoming these challenges. The Healthy China 2030 will inform all aspects of government policy and emphasize innovation, scientific development, justice, and fairness. It is aimed at advocating lifelong learning, encouraging senior social engagement, and boosting elderly heath. The government has substantially increased its investment in geriatric research. Many international exchange programs and collaborative projects are being conducted between major hospitals and universities worldwide and international organizations, such as the World Health Organization (WHO) [8]. Geriatric medicine is emerging as one of the most advanced and appreciated subspecialties in China. There are both great challenges and opportunities ahead for people working in the field of geriatric medicine. We look forward to the common development of geriatrics to provide more and better services for the elderly in China.
